# Délais de prise en charge des syndromes coronariens aigus avec sus-décalage du segment ST à Ouagadougou et facteurs associés à un allongement de ces délais: étude transversale à propos de 43 cas colligés au CHU-Yalgado Ouédraogo

**Published:** 2012-12-30

**Authors:** Nobila Valentin Yameogo, André Samadoulougou, Georges Millogo, Koudougou Jonas Kologo, Karim Kombassere, Boubacar Jean Yves Toguyeni, Patrice Zabsonre

**Affiliations:** 1 CHU-Yalgado OUEDRAOGO, Service de cardiologie (Professeur Patrice ZABSONRE), 03BP7022 Ouagadougou 03, Burkina Faso

**Keywords:** Syndrome coronarien, prise en charge, délai, Burkina Faso, coronary syndrome, management, delay, Burkina Faso

## Abstract

La prise en charge de l'infarctus du myocarde est une course contre la montre et les trois premières heures constituent les « golden hours ». Les objectifs de ce travail étaient de déterminer le délai de prise en charge des infarctus du myocarde du myocarde au Burkina Faso, les facteurs liés à un allongement du délai et le pronostic des patients. Il s'agit d'une étude transversale descriptive menée de Septembre 2010 à Août 2011. Le critère d'inclusion était l'infarctus du myocarde dont le diagnostic était basé sur des critères clinique (douleur angineuse), électrocardiographique (sus-décalage persistant du segment ST dans au moins deux dérivations contiguës du même territoire coronaire, onde Q de nécrose) et biologique (élévation de la troponine). Les informations relatives au délai de prise en charge ont été recueillies: début du premier symptôme, contact avec le premier agent de santé et le cardiologue, nombre de centre de santé consulté avant le transfert en cardiologie, situation géographique des patients, moyen de transport utilisé. Les données ont été analysées grâce au logiciel SPSS version 17. Durant la période d’étude, 43 patients d’âge moyen de 56,51 ± 12,91 ans ont été admis pour infarctus du myocarde. Plus de la moitié des patients (72,0%) habitait Ouagadougou et sa banlieue. Le délai moyen entre le début de la douleur et la consultation dans la première structure sanitaire était de 48 ± 20,8 heures; celui entre le début de la douleur et la réalisation du premier ECG était en moyenne de 8,6 ±4,5 jours. Le délai entre la réalisation de l'ECG et l'admission dans le service de cardiologie était de 4,35 ±4,0 jours [00 heure et 13 jours]. Le délai entre l'admission dans le service de cardiologie et la thrombolyse était de 34 minutes. Enfin le délai entre le début de la douleur et le contact avec le cardiologue était de 9,6±3,5 jours. Il n'y avait pas de différence statiquement significative (P = 0,076) entre le délai de consultation des malades résidant en campagne et ceux résidant en ville. Le moyen de déplacement le plus utilisé était les transports en commun (67,4%). Aucun patient n’était référé par un transport médicalisé. Aucun patient n’était référé après la première consultation dans une structure sanitaire. Seuls 6 patients étaient référés avec le diagnostic de SCA ST+. L’âge de plus de 60 ans (P = 0,016), la prescription des antalgiques (p =0,021) et le niveau socioéconomique faible (P= 0,038) étaient également des facteurs associés à un allongement des délais de prise en charge. La reperfusion myocardique était constituée par la thrombolyse à la streptokinase qui a été réalisée chez 2 patients. La coronarographie n'a pas été réalisée. L’évolution a été marquée par 5 décès (11,6%). la prise en charge des infarctus du myocarde au Burkina Faso est caractérisée par de très longs délais. Elle n'est de ce fait pas optimale.

## Introduction

Les syndromes coronariens aigus avec sus-décalage persistant du segment ST (SCA ST+) constituent un problème majeur de santé publique de par leur prévalence et leur mortalité. En effet, tout patient à la phase aiguë d'un infarctus du myocarde est en danger de mort immédiate. Plus du tiers des décès vont survenir dans la première heure et la moitié au cours des 24 premières heures. Le pronostic est fonction du temps mis entre le début des symptômes et celui de la prise en charge appropriée [1–4]. La nécrose myocardique s'accentue dans le temps et tout l'objectif de la prise en charge est de limiter la nécrose [5]. Cette étude avait pour objectifs de déterminer le délai moyen de prise en charge des infarctus du myocarde, les facteurs liés à un allongement du délai et le pronostic des patients au CHU-Yalgado Ouédraogo.

## Méthodes

Nous avons réalisé une étude transversale de Septembre 2010 à Août 2011 incluant tous les patients admis dans le service de cardiologie du CHU-YO pour infarctus du myocarde. Le diagnostic d'infarctus était basé sur la clinique (douleur angineuse), l’électrocardiogramme (sus-décalage persistant du segment ST dans au moins deux dérivations contiguës du même territoire coronaire, onde Q de nécrose), la troponinémie et les CPK-MB (élévation de la troponine et des CPK-MB). Un questionnaire a permis de recueillir les informations relatives au délai de prise en charge: début du premier symptôme, contact avec le premier agent de santé et le cardiologue, nombre de centre de santé consulté avant le transfert en cardiologie, composition du premier traitement administré, situation géographique des patients, moyen de transport utilisé. Nous avons réalisé un examen clinique complet à la recherche des facteurs de risque cardio-vasculaire et d’éventuelles complications. La biologie (glycémie, NFS, Créatininémie, Uricémie, Troponinémie, CPK-MB), l'ECG de surface et l’échographie cardiaque étaient systématiques. Les données ont été analysées grâce au logiciel SPSS version 17 pour windows.

## Résultats

Nous avons inclus 43 patients dont 38 hommes. L’âge moyen des patients était de 56,51 ± 12,91 ans (23 et 90). Les caractéristiques générales des patients sont résumées dans le Tableau 1. Plus de la moitié des patients (72,0%) habitait Ouagadougou et sa banlieue. Le motif de consultation était la douleur thoracique dans tous les cas. A l'admission, 4 patients présentaient une insuffisance cardiaque. L'ECG et les marqueurs biologiques étaient contributifs dans tous les cas.

**Tableau 1:** Caractéristiques générales des 43 patients hospitalisés au CHU-YO de Septembre 2010 à Août 2011 pour syndromes coronariens aigus avec sus-décalage persistant du segment ST (SCA ST+)

Caractéristique	Nombre	Pourcentage
Nombre	43	100
Sex-ratio (38H/5F) = 7		
Age moyen = 56,51 ± 12,91 ans (23 et 90)		
HTA	26	60,5
Surcharge pondérale/Obésité	14	32,5
Sédentarité	11	25,5
Tabagisme actif	13	30,2
Diabète	6	13,9
Dyslipidémie	9	20,9
HIV+	3	6,9
Territoire antérieur	29	67,4
Atteinte du ventricule droit	2	4,6

### Les délais

**Figure 1:**
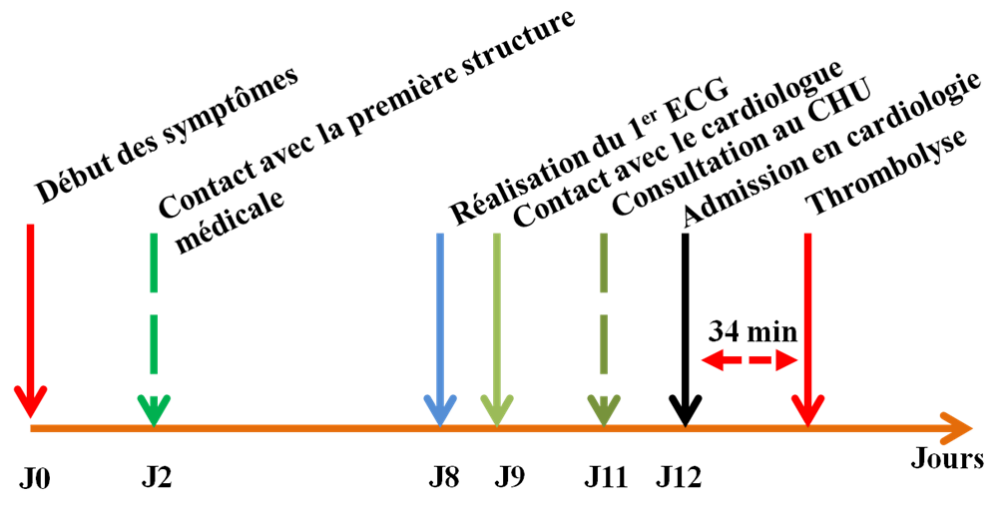
Délais en jour (J) de prise en charge des syndromes coronariens avec sus décalage du segment ST

Le délai moyen entre le début de la douleur et la consultation dans la première structure sanitaire était de 48 ± 20,8 heures (1 et 72 heures). Le délai entre le début de la douleur et la réalisation du premier ECG était en moyenne de 8,6 ±4,5 jours (90 minutes et 18 jours). Le délai moyen de consultation au CHU-YO était de 11 ± 7,5 jours avec des extrêmes de 4 heures et 29 jours. Seuls 4 patients étaient admis avant la 12ème heure. Ces patients ont consulté directement au CHU-YO. Le délai entre la réalisation de l'ECG et l'admission dans le service de cardiologie était de 4,35 ± 4,0 jours [00 heure et 13 jours]. Le délai entre l'admission dans le service de cardiologie et la thrombolyse était de 34 minutes. Enfin le délai entre le début de la douleur et le contact avec le cardiologue était de 9,6±3,5 jours (30 minutes et 21 jours). Ces délais sont résumés dans la Figure 1.

### Facteurs liés aux délais

La situation géographique des patients: Il n'y avait pas de différence statiquement significative (P = 0,076) entre le délai de consultation des malades résidant en campagne et ceux résidant en ville. Le moyen de déplacement le plus utilisé était les transports en commun (67,4%). Aucun patient n’était référé par un transport médicalisé. Le traitement prescrit dans les premières structures sanitaires avant la consultation cardiologique était les pansements gastriques (76,4%), les AINS (72,0%) et les antalgiques de palier I (69,7%).

Le circuit de prise en charge: le nombre de structure sanitaire consultée par les patients était en moyenne de 3. Ce nombre n’était pas significativement différent entre les patients vivant en milieu urbain et ceux vivant en campagne (p = 0,45).

Le manque de référence: aucun patient n’était référé après la première consultation dans une structure sanitaire. Dans plus de la moitié des cas (58,1%) les patients avaient changé de structure de soins de par leur propre chef. Les erreurs diagnostiques: seuls 6 patients étaient référés avec le diagnostic de SCA ST+. L’âge de plus de 60 ans (P = 0,016), la prescription des antalgiques (p =0,021) et le niveau socioéconomique faible (P= 0,038) étaient également des facteurs d'allongement des délais.

### Le traitement

Le traitement était constitué par la thrombolyse à la streptokinase chez 2 patients, les héparines de bas poids moléculaire dans 86,0% des cas, les bêtabloquants dans 79,0% des cas, l'aspirine et la statine dans tous les cas, les IEC dans 88,3% des cas et les dérivés nitrés dans 51,1% des cas. La dobutamine et les diurétiques de l'anse ont été utilisés chez 7 patients. Aucune angioplastie n'a été réalisée. L’évolution a été marquée par 5 décès (11,6%). Les différentes complications sont résumées dans le Tableau 2. La durée moyenne d'hospitalisation était de 14,3±9,1 jours (1 et 42 jours).

**Tableau 2:** Complications de 43 syndromes coronariens aigus avec sus-décalage persistant du segment ST (SCA ST+) hospitalisés dans le service de cardiologie du CHU-YO de Septembre 2010 à Août 2011

Complication	Nombre	Pourcentage
Insuffisance cardiaque	7	16,2
Trouble du rythme	4	9,3
Anévrisme du ventricule gauche	3	6,ç
Péricardite	2	4,6
Hémorragie cérébrale	1	2,3

## Discussion

Le pronostic d'un SCA ST+ dépend de la précocité de la reperfusion coronaire par thrombolyse ou angioplastie primaire. L'objectif de la prise en charge à la phase aiguë est de réduire la taille de l'infarctus par une intervention précoce et d'améliorer ainsi son pronostic en réduisant le risque de mortalité hospitalière et à un an. La relation étroite entre des délais courts de reperfusion de l'artère responsable de l'infarctus ST+ et une réduction de mortalité est documentée par de nombreuses études [1–6]. Les étapes de la prise en charge des SCA ST+ sont nombreuses. Plusieurs délais sont utilisés dans la littérature anglaise: time to reperfusion, door-to-balloon (D2B), symptom onset-to-door, time-to-first call. La société européenne de cardiologie a proposé la notion de « premier contact médical » (PCM), défini par « le lieu (ambulance ou hopital) où, au moins en principe, le traitement de reperfusion pourrait être initié » (the first medical contact is the place (ambulance or hospital) where, at least in principle, reperfusion therapy could be given).

Selon les recommandations européennes de 2008 [7], tout patient pris en charge dans les 12 premières heures suivant le début des symptômes doit être reperfusé. Entre 12 et 24 h, la discussion se fera « au cas par cas » en fonction de la clinique (douleur persistante, état hémodynamique) et de l'ECG à l'admission (onde Q de nécrose constituée, persistance d'un sus-décalage du segment ST, étendue de l'infarctus). Au-delà de 24 h, il n'y a pas de bénéfice à rouvrir une artère occluse chez un patient stable.

La stratégie de reperfusion chez un patient pris en charge précocement repose sur l'estimation du délai « PCM-inflation du ballon ». S'il est inférieur à deux heures (90 minutes si la prise en charge est précoce dans les deux premières heures suivant le début de la douleur, l'infarctus étendu et le risque hémorragique faible), une angioplastie primaire est réalisée. Dans le cas contraire une fibrinolyse est décidée après évaluation du risque hémorragique. Dans les dernières recommandations de la société européenne de cardiologie sur la revascularisation myocardique publiées en 2010, le même délai « PCM-inflation du ballon » de deux heures est proposé, 90 minutes pour les patients de moins de 75 ans ayant un infarctus du myocarde étendu et un début des symptômes récent [8]. Le registre français FAST MI (564 patients traités par angioplastie primaire en octobre 2005) rapporte un délai médian « début des symptômes-premier appel » de 75 minutes suivi d'un délai « 1er appel - ponction artérielle » de 165 minutes [8].

Au Burkina Faso, il n'existe pas de stratégie ni de circuit de prise en charge des SCA ST+. Les délais définis par les sociétés savantes sont encore illusoires. Les Facteurs associés à un allongement des délais de prise en charge dans notre contexte sont liés au manque de référence et aux erreurs diagnostiques essentiellement. L’âge de plus de 60, la prescription des antalgiques et le niveau socioéconomique faible étaient également des facteurs d'allongement des délais. Les difficultés diagnostiques et les « Circuits de prise en charge » et l’âge avancé contribuent à allonger le délai de l'angioplastie en Europe [5, 10–12].

## Conclusion

La quasi-totalité des patients victimes d'un syndrome coronarien aigu ST+ sont pris en charge hors délai. Ce constat est le témoin d'un manque de stratégie nationale de prise en charge de cette pathologie. Les facteurs associés à un allongement des délais de prise en charge sont liés au manque de référence et aux erreurs diagnostiques. L'absence de la coronarographie rend toujours incomplète la prise en charge.
